# Characterization of Patients With Obstructive Sleep Apnea Syndrome Undergoing Drug-Induced Sleep Endoscopy: A Retrospective Study of Nine Years (2014-2022)

**DOI:** 10.7759/cureus.78811

**Published:** 2025-02-10

**Authors:** Margarida Araújo Martins, Catarina Lombo, Clara Mota, Catarina Pinto, Rita Moura, Rui Fonseca

**Affiliations:** 1 Otolaryngology - Head and Neck Surgery, Hospital da Senhora da Oliveira, Guimarães, PRT; 2 Otorhinolaryngology, Centro Hospitalar Tâmega e Sousa, Penafiel, PRT; 3 Otorhinolaryngology, Hospital da Senhora da Oliveira, Guimarães, PRT

**Keywords:** apnea hypopnea index (ahi), dise, drug induced sleep endoscopy, obstructive sleep apnea, upper way obstruction, vote classification

## Abstract

Introduction: Obstructive sleep apnea syndrome (OSAS) is the most prevalent sleep-related breathing disorder associated with an increased risk of cardiovascular and cerebrovascular complications. Identifying the anatomical sites of airway obstruction is crucial for optimizing treatment, particularly in patients requiring surgical intervention. In recent years, drug-induced sleep endoscopy (DISE) has emerged as a valuable diagnostic tool for evaluating upper airway obstruction in OSAS patients.

Objectives: To characterize the OSAS population undergoing DISE and correlate endoscopic findings with patient characteristics and obstruction sites.

Materials and methods: A retrospective study was conducted by reviewing medical records of patients who underwent DISE between 2014 and 2022 at Hospital da Senhora da Oliveira, Guimarães. The analyzed variables included age, gender, body mass index (BMI), apnea-hypopnea index (AHI), and the level and pattern of obstruction based on the velum, oropharynx, tongue, and epiglottis (VOTE) classification. Statistical analysis was performed using IBM SPSS Statistics v27.0 (IBM Corp., Armonk, USA).

Results: A total of 71 patients underwent DISE, with ages ranging from 25 to 68 years. The majority were male (84%, n=60). Regarding BMI distribution, 18.3% (n=13) of patients were classified as obese, 67.6% (n=48) as overweight, and 14.1% (n=10) had a normal weight. Most patients had moderate OSAS (56.3%, n=40), followed by severe (26.8%, n=19) and mild OSAS (16.9%, n=12). A significant positive correlation was observed between AHI and BMI (p=0.028). Multilevel obstruction was identified in 75% of patients, with the most common collapse site being the soft palate, followed by the oropharynx, base of the tongue, and epiglottis. No statistically significant differences were found in the obstruction site, pattern, or severity based on AHI or BMI. However, at the palatal level, a positive trend was observed between AHI and obstruction severity (r=0.153; p>0.05).

Conclusions: DISE is a safe and reliable diagnostic modality for identifying upper airway collapse sites, revealing anatomical alterations that may not be apparent during standard physical examination. This technique plays a crucial role in evaluating patients considered for surgical intervention or those who have not responded to first-line therapies. DISE enables a personalized approach to OSAS management by tailoring treatment strategies to individual patient anatomy and obstruction patterns.

## Introduction

Obstructive sleep apnea syndrome (OSAS) is the most prevalent sleep disorder, with recent population studies indicating that 13-50% of men and 6-23% of women over 50 have moderate to severe OSAS [1,2]. It is characterized by recurrent episodes of partial or complete upper airway collapse during sleep, leading to oxygen desaturation and sleep fragmentation. This results in daytime sleepiness, reduced quality of life, and increased cardiovascular risk [3].

While continuous positive airway pressure (CPAP) is the gold standard treatment, 30-50% of patients have poor adherence [4]. Thus, investigating the causes of CPAP failure and exploring other treatment options like upper airway surgery or mandibular advancement devices is crucial [5].

Polysomnography diagnoses OSAS but provides no information about airway anatomy or collapse patterns - key factors in treatment decisions, especially when considering CPAP alternatives [6]. To this end, Müller's maneuver (MM) was introduced, allowing airway evaluation while the patient is awake. However, MM has the limitation of not allowing patient evaluation during sleep, when muscle tone is lower and airway physiology is different. In 1991, drug-induced sleep endoscopy (DISE) was introduced by Pringle and Croft [7] to allow airway evaluation during a sedated state mimicking sleep.

In the past two decades, DISE has emerged as a method for three-dimensional evaluation of the upper airway during pharmacologically induced sleep. Numerous studies have sought to establish the efficacy of DISE as a diagnostic tool for sleep apnea, demonstrating that the procedure is minimally invasive, safe, and cost-effective [8]. It has already been demonstrated that surgical planning based on awake examination findings can be significantly altered following DISE in over 50% of cases. These alterations are most frequently associated with hypopharyngeal and laryngeal structures. However, it is important to note that these differences in findings do not necessarily translate to improved surgical success rates [8]. The Otorhinolaryngology Department of Hospital da Senhora da Oliveira in Guimarães introduced this examination in 2014 for the evaluation of some patients in the specific adult OSAS consultation. This study aims to characterize the OSAS population undergoing DISE at our institution from 2014-2022 and to relate this to the characteristics and site of upper airway obstruction observed.

## Materials and methods

We reviewed records of OSAS patients who underwent DISE from 2014-2022, collecting demographic and anthropometric data (sex, age, body mass index (BMI)), OSAS severity, physical exam findings, and airway collapse degree and pattern on DISE.

OSAS diagnosis was confirmed by polysomnography and classified as mild (apnea-hypopnea index (AHI) 5-15 with symptoms), moderate (AHI 15-30), or severe (AHI>30) [9].

DISE was performed in the following situations: when there were doubts about the upper airway collapse site; when alternative treatments to CPAP were being considered; after CPAP treatment failure, trying to understand the reason for failure and evaluate anatomical alterations amenable to surgical correction; and in cases of upper airway surgery failure, such as palatoplasties performed without prior DISE (failure considered when there is an AHI reduction <50% or AHI>20) [10].

**Figure 1:**
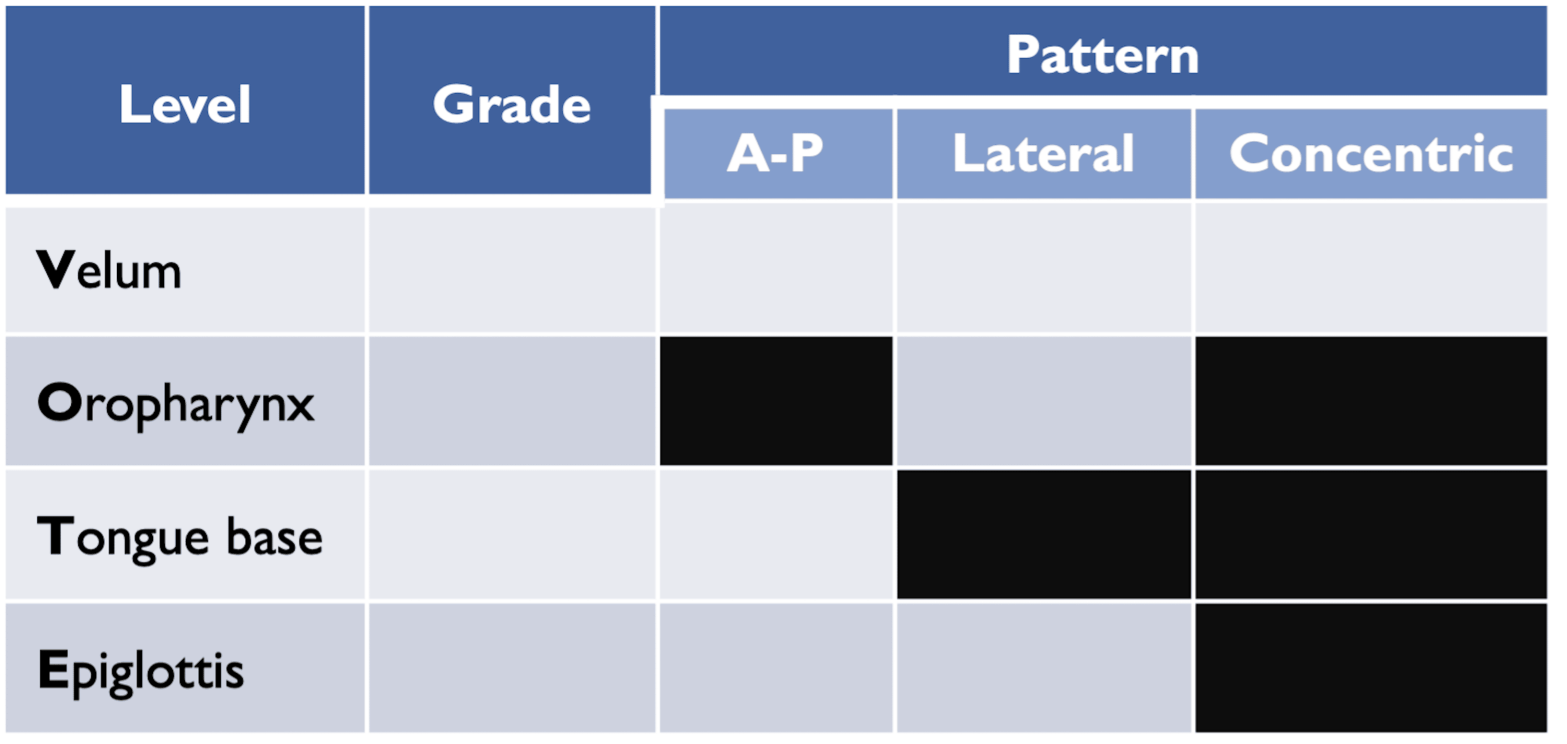
VOTE classification (i) The degree was mentioned according to the scale of 0-25%, 25-50%, 50-75%, and 75-100%. (ii) The pattern was registered when the degree of collapse was greater than 0. A-P: anteroposterior; VOTE: velum, oropharynx, tongue, and epiglottis

DISE was conducted under sedation, in the supine position, in the operating room. Dexmedetomidine infusion was used for sedation, with a loading dose of 0.5-1 µg/kg for 10 minutes, followed by a maintenance dose of 0.2-1 µg/kg/hour, titrated to effect, with bispectral index (BIS) oscillating between 50-70. During each procedure, between two to six apneas were observed. The results obtained were recorded according to the velum, oropharynx, tongue, and epiglottis (VOTE) classification (Figure 1), and for each obstruction level, the obstruction pattern was recorded as anteroposterior, concentric, or lateral, and the degree of obstruction was recorded using the scale 0-25%, 25-50%, 50-75%, and 75-100% [11]. Collapse was considered when the degree of obstruction was greater than 25%.

All procedures were performed or supervised by the same physician. Categorical variables were described as absolute numbers and frequencies, while continuous variables were presented as means (standard deviation). Statistical analysis was performed using IBM SPSS Statistics v27.0 (IBM Corp., Armonk, USA). The Mann-Whitney U test, with a 95% confidence interval, was employed to compare two groups of variables. Relationships between DISE findings and anthropometric data with AHI scores were assessed using the Spearman correlation coefficient. Statistical significance was set at p<0.05.

## Results

During the studied period, 71 DISEs were performed. The mean age of the evaluated patients was 48.3 ± 8.7 years, and the mean BMI was 27.6 ± 2.6 kg/m^2^. The majority of the patients were male (84.5 %) in a ratio of 5:1 compared to female patients (Table 1).

**Table 1:** Demographic data of the study population BMI: body mass index; AHI: apnea-hypopnea index

Variables		n (%)	Mean ± SD	Range
Gender	Male	60 (84.5%)		
	Female	11 (15.5%)		
Age (years)			48.3 ± 8.7	25 - 68
BMI (kg/m^2^)			27.6 ± 2.6	22.7 - 37.7
AHI (events/hour)			26.4 ± 16.1	5.7 - 94.2

**Figure 2:**
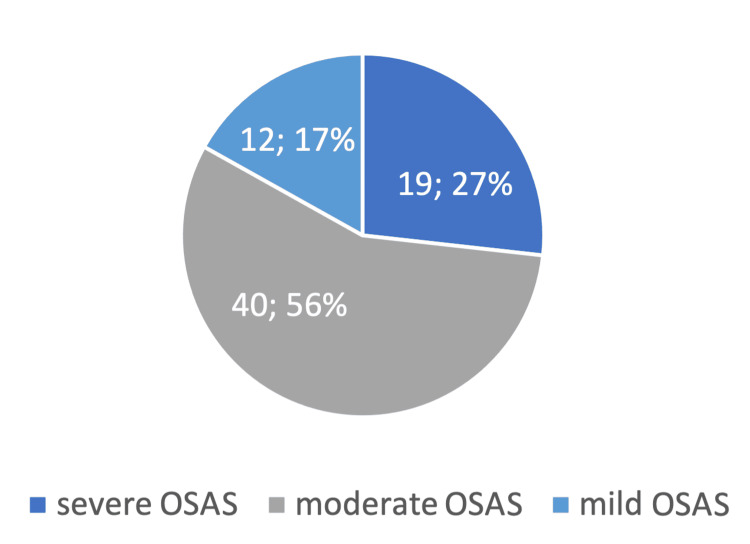
OSAS classification of the study population (N=71) The values represent n; %. OSAS: obstructive sleep apnea syndrome

The mean AHI of patients who underwent DISE was 26.4 ± 16.1 events/hour, with the majority having moderate OSAS (n=40), followed by severe OSAS (n=19), while 12 had mild OSAS (Figure 2).

**Figure 3:**
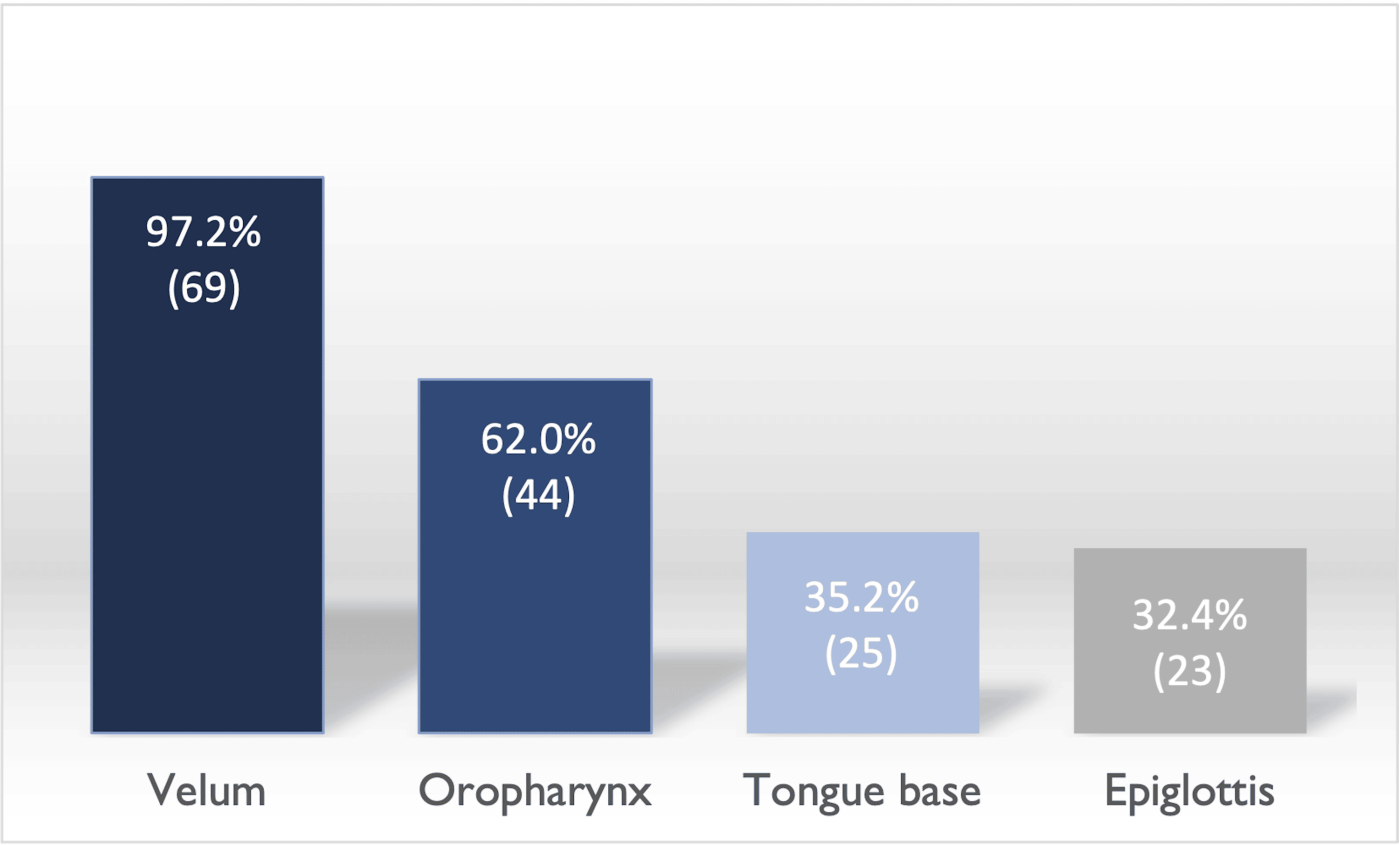
Site of collapse in DISE The values are presented as % (n). DISE: drug-induced sleep endoscopy

**Figure 4:**
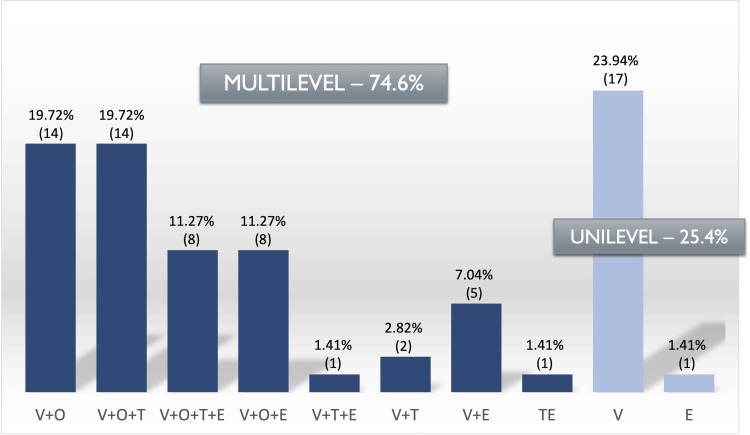
Collapse level in DISE The values are presented as % (n). Dark blue bars indicate multilevel and light blue bars indicate unilevel obstruction. V: velum, O: oropharynx, T: tongue, E: epiglottis (VOTE classification); DISE: drug-induced sleep endoscopy

**Figure 5:**
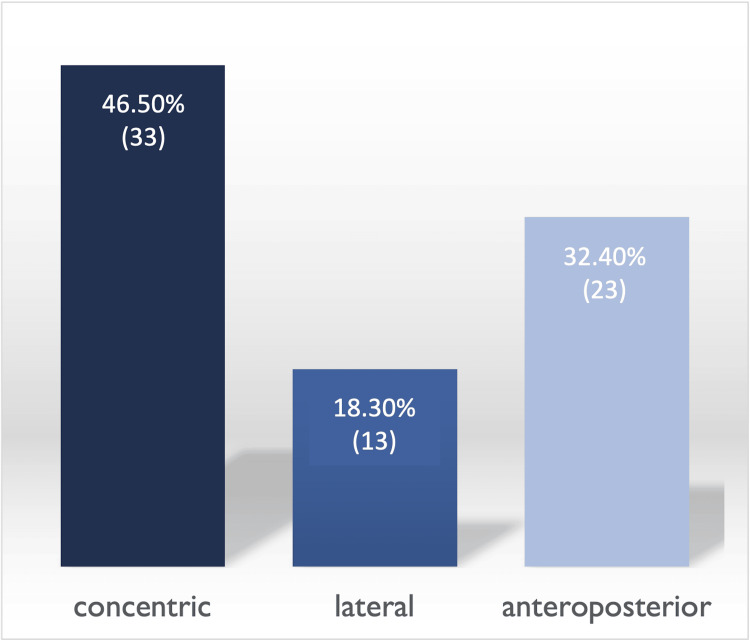
Velum type of collapse The values are presented as % (n).

Regarding the collapse level in DISE, in most cases, it was present at the velopharynx level (97%), followed by the oropharynx (62%), tongue base (35%), and epiglottis (32%) (Figure 3). We observed multilevel obstruction in the vast majority of patients undergoing DISE (74.6%) (Figure 4). The type of collapse observed at the level of the velopharynx (46.5%) was mainly concentric, but also anteroposterior (32.4%) and lateral (18.3%) (Figure 5).

Regarding the reason for performing DISE, the majority (68%, n=48) were performed due to doubts about the upper airway collapse site, 18 were performed to understand the reason for poor CPAP adaptation, and five were performed after surgical treatment failure. Of the patients who did not respond to surgery, two had collapse at the epiglottis level, another two had posterior tongue base fall, and another had grade III Friedman lingual tonsil hypertrophy [12], subsequently undergoing lingual tonsillectomy.

**Figure 6:**
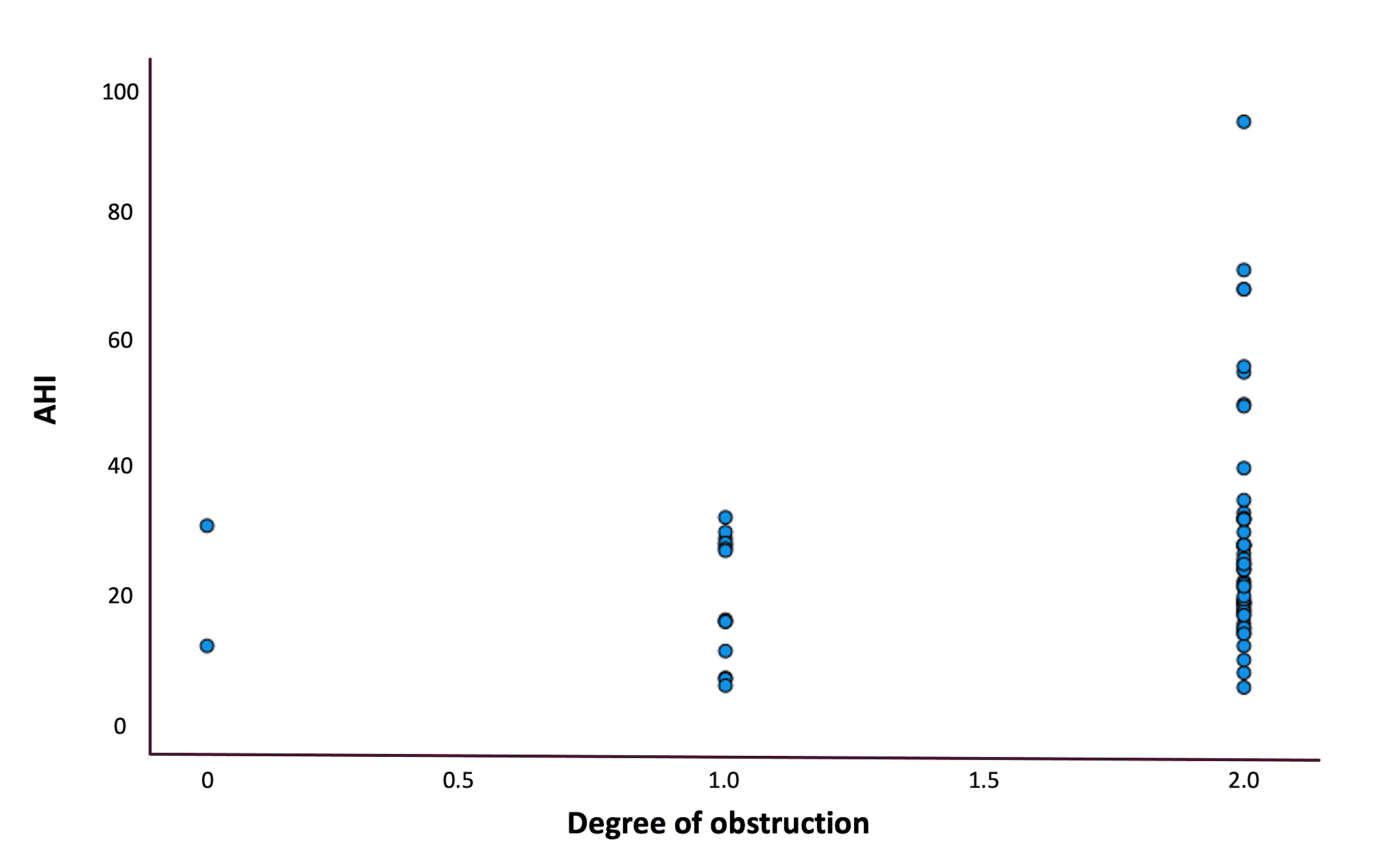
Spearman correlation between AHI and degree of collapse of the velopharynx (r=0.253; p>0.05) Degree of obstruction of the velopharynx: no obstruction (0, <50%), partial obstruction (1, 50%-75%), and complete obstruction (2, >75%). AHI: apnea-hypopnea index

As expected, there was a positive correlation between AHI and BMI (p=0.028). We did not find significant differences in the site, pattern, or degree of obstruction according to patients' AHI or BMI. However, at the palatal level, we observed a positive trend between AHI and the degree of obstruction (r=0.253; p>0.05) (Figure 6).

## Discussion

The diagnosis and management of OSAS have evolved significantly over recent decades. While polysomnography remains the gold standard for OSAS diagnosis [6], evaluation of upper airway anatomy is crucial as the pathophysiology of the disease occurs at this level. In some patients, the anatomical cause of collapse is evident during awake upper airway examination; however, in others, it remains unclear [13].

DISE has gained relevance in clinical practice as it allows for observation of the upper airway and collapse sites during a pharmacologically induced state that mimics natural sleep. The choice of drugs for this purpose has been debated, with propofol and midazolam combination being the most common. In our department, we used dexmedetomidine, an α2-adrenergic receptor agonist, which allows for a more natural sleep architecture with fewer respiratory depressant effects [14].

DISE has several limitations, including the potential for drug-induced excessive sedation to induce false obstruction [15] and its inability to capture more than a brief segment of the night's sleep, thereby precluding the replication of rapid eye movement (REM) sleep stages [13]. Additionally, DISE is an expensive examination requiring specialized equipment and personnel [16], making it not feasible to be performed on all patients with OSAS. It is indicated when alternative treatments to CPAP are considered and when there is a total or partial failure of the instituted treatment, according to the European Position Paper on DISE [16].

In our department, DISE was conducted whenever the objective examination raised uncertainties regarding upper airway surgery or mandibular advancement devices. The DISE findings were employed to determine the type of surgical intervention. So, if we had a predominantly lateral collapse at the velopharyngeal and oropharyngeal levels, we performed lateral pharyngoplasty techniques, and if we had a predominantly anteroposterior collapse, a uvulopalatopharyngoplasty using radiofrequency was done. The DISE procedures performed in the context of surgical failure indeed allowed for understanding the cause of failure and facilitated the modification of treatment or the implementation of other types of interventions, such as lingual tonsillectomy. This underscores the importance of conducting DISE in the preoperative period. These findings are consistent with existing literature, which suggests that the epiglottis and the base of the tongue are the most commonly overlooked sites of obstruction during objective assessment in awake patients [8,17]. In our study, the most frequent collapse site observed during DISE was the velopharynx (97%), followed by the oropharynx (62%), tongue base (35%), and epiglottis (32%), with multilevel collapse in 75% of cases. These results align with other studies involving a larger number of patients, notably Vroegop et al. who reported palatal collapse in 81% of cases with multilevel collapse occurring in 68.2% of cases in a sample of 1249 patients [18], and Pilaete et al. who found a palatal obstruction in 93.4% of cases with multilevel obstruction in 78.8% of cases in a sample of 100 patients [19]. On the other hand, Salamanca et al. described the presence of oropharyngeal obstruction in 96% of cases in a sample of 641 patients [20]. This discrepancy is attributed to a different classification method for DISE findings, where the nose, oropharynx, hypopharynx, and larynx (NOHL) classification was employed [9]. This underscores the importance of adopting a simple and unified classification system for describing DISE results to standardize studies.

The high male-to-female ratio (5:1) in our sample reflects the different prevalence of OSAS in the general population according to gender. This pathology is more common in male patients, particularly those below 60 years of age, due to hormonal differences and body fat distribution. Generally, women exhibit a smaller neck circumference than men of the same age and BMI [1].

We could not find significant differences in the site, pattern, or degree of obstruction according to patients' AHI or BMI. However, at the palatal level, we observed a positive trend between AHI and the degree of obstruction. Therefore, although not entirely accurate, the patient's AHI can be extrapolated by assessing the degree of obstruction at this level. Lim et al. proposed that when a soft palate is suspected in OSAS, computed tomography measurement of soft palate length is a valid method for estimating the degree of velum obstruction and the severity of OSAS [21].

Our study has limitations, including a small sample size and a lack of assessment of DISE's impact on surgical success. However, it highlights the importance of DISE in OSAS evaluation in otorhinolaryngology, proving useful especially when there are doubts in patient assessment.

## Conclusions

OSAS is a complex disease with multiple anatomical and systemic contributing factors. Determining upper airway topography is essential in evaluating OSAS patients. DISE can highlight the degree of collapse observed during an awake examination and sometimes reveal collapse sites that may go unnoticed. Therefore, DISE is crucial for patients considering surgical treatment or those whose treatment has failed. The present study demonstrated that BMI and DISE findings, particularly velopharyngeal collapse, are significant factors associated with OSAS severity. These findings may contribute to a better understanding of key mechanisms underlying OSAS pathophysiology. Additional studies employing larger sample sizes and a standardized, unified classification system are necessary to comprehensively evaluate the impact of DISE on surgical success rates and overall patient outcomes.
